# Biotic and Abiotic Properties Mediating Plant Diversity Effects on Soil Microbial Communities in an Experimental Grassland

**DOI:** 10.1371/journal.pone.0096182

**Published:** 2014-05-09

**Authors:** Markus Lange, Maike Habekost, Nico Eisenhauer, Christiane Roscher, Holger Bessler, Christof Engels, Yvonne Oelmann, Stefan Scheu, Wolfgang Wilcke, Ernst-Detlef Schulze, Gerd Gleixner

**Affiliations:** 1 Max Planck Institute for Biogeochemistry, Jena, Germany; 2 Institute of Ecology, Friedrich- Schiller-University Jena, Jena, Germany; 3 UFZ, Helmholtz Centre for Environmental Research, Department of Community Ecology, Halle, Germany; 4 Department of Plant Nutrition, Faculty of Agriculture and Horticulture, Humboldt-Universität zu Berlin, Berlin, Germany; 5 Geoecology/Geography, Eberhard Karls University of Tübingen, Tübingen, Germany; 6 J.F. Blumenbach Institute of Zoology and Anthropology, Georg August University Göttingen, Göttingen, Germany; 7 Soil Science Group, Geographic Institute, University of Berne, Bern, Switzerland; Graz University of Technology (TU Graz), Austria

## Abstract

Plant diversity drives changes in the soil microbial community which may result in alterations in ecosystem functions. However, the governing factors between the composition of soil microbial communities and plant diversity are not well understood. We investigated the impact of plant diversity (plant species richness and functional group richness) and plant functional group identity on soil microbial biomass and soil microbial community structure in experimental grassland ecosystems. Total microbial biomass and community structure were determined by phospholipid fatty acid (PLFA) analysis. The diversity gradient covered 1, 2, 4, 8, 16 and 60 plant species and 1, 2, 3 and 4 plant functional groups (grasses, legumes, small herbs and tall herbs). In May 2007, soil samples were taken from experimental plots and from nearby fields and meadows. Beside soil texture, plant species richness was the main driver of soil microbial biomass. Structural equation modeling revealed that the positive plant diversity effect was mainly mediated by higher leaf area index resulting in higher soil moisture in the top soil layer. The fungal-to-bacterial biomass ratio was positively affected by plant functional group richness and negatively by the presence of legumes. Bacteria were more closely related to abiotic differences caused by plant diversity, while fungi were more affected by plant-derived organic matter inputs. We found diverse plant communities promoted faster transition of soil microbial communities typical for arable land towards grassland communities. Although some mechanisms underlying the plant diversity effect on soil microorganisms could be identified, future studies have to determine plant traits shaping soil microbial community structure. We suspect differences in root traits among different plant communities, such as root turnover rates and chemical composition of root exudates, to structure soil microbial communities.

## Introduction

The soil microbial community holds a central position in ecosystem processes like carbon and nitrogen cycling (e.g., [1], [2]). The performance and shape of soil microbial communities on one hand depend on soil properties, such as pH, temperature, texture and moisture [3]–[6], but on the other hand the soil microbial community is closely linked to plant communities through complex interactions [7]–[12]. Plants affect the soil microbial community through biomass production, litter quality, seasonal variability of litter production, root-shoot carbon allocation and root exudates [13]–[15]. In turn, soil microbial communities mineralize organic matter and enhance nutrient release by mineral weathering. Both processes increase the availability of nutrients enhancing plant growth [16], [17] and consequently accelerate the matter flow between the aboveground and belowground parts of ecosystems.

Plant diversity influences a wide range of ecosystem processes, but the underlying mechanisms are not well understood [18]; for example the link between plant diversity and belowground processes is just fragmentarily explained. Increasing plant diversity modifies resource availability for microbial communities in soil [19], [20], which might lead to higher niche differentiation and facilitation of the soil microbial community [21]. Beside species richness, the number of plant functional groups, i.e. species with similar morphological, phenological and physiological traits, impact soil microorganisms [21]. Plant functional groups, such as legumes and grasses, differ in litter quality and the amount of carbon and nitrogen released to the soil [22], [23], thereby affecting microbial decomposition processes [24]. Bacteria and fungi form most of the soil microbial biomass and represent the main drivers of organic matter turnover [25]. Since both groups prefer different qualities of resources they might be differently affected by plant diversity and plant community composition. Fungi are able to decompose litter with high C:N ratios [26]. Therefore, it has been suggested that plant communities providing litter with high C:N ratio favor decomposition by fungi, whereas plant communities producing litter with low C:N ratio favor decomposition by bacteria [27]. Moreover, there are differences among bacteria: Gram-negative bacteria are mainly root-associated and thus decompose organic molecules of low molecular weight [28], whereas Gram-positive bacteria are decomposing more complex materials, such as soil organic matter and litter [29]. As a consequence, the presence of certain plant functional groups is likely to promote distinct microbial groups. Therefore, higher plant diversity, as number of species or number of functional groups, might affect the composition of the soil microbial community by differences in litter input quantity and quality. Most studies investigating effects of plant diversity on soil microbial community focus on plant-originated inputs, often ignoring that differences in diversity and composition of plant communities also affect microclimatic conditions such as soil moisture. Conversely, studies considering plant mediated effects on soil moisture [23], [30] usually do not account for root inputs or changes in the soil microbial composition. Identifying the relative importance of drivers changing soil microbial communities is needed to better understand the functioning of soils [31].

We assessed the effect of plant diversity and functional group composition on soil microbial communities using phospholipid fatty acids (PLFAs) [29], [32], [33]. The study was conducted in the framework of the Jena Experiment, a biodiversity experiment established by sowing different combinations of grassland species [34] on a fallow agricultural soil. In addition to experimental plots with different levels of plant diversity and vegetation-free bare ground plots, we studied long-term meadows and on-going arable plots as adjacent to the field experiment as control sites to assessed, how the soil microbial community developed five years after establishing the experimental site. We hypothesized that (1) higher plant diversity increases soil microbial biomass, caused by higher amounts of litter input as well as by improved microclimatic conditions for soil microbes, and (2) plant functional group composition drives composition of the soil microbial community, exemplified e.g., by changes of fungal-to-bacterial biomass ratio (F:B ratio). Due to the production of low quality litter, we expected plant communities containing grasses but not legumes to favor fungi, whereas plant communities producing litter of high quality to favor bacteria.

## Materials and Methods

### Site description and experimental design

The field site of the Jena Experiment is located close to the city of Jena (Germany) in the floodplain of the river Saale (50°55′ N, 11°35′ E, 130 m a.s.l.). No specific permission was required to work on “The Jena Experiment” and no endangered or protected species were involved in this study. The soil (Eutric Fluvisol) has developed from up to 2 m thick fluvial sediments presenting a systematic variation of soil texture. The sand content decreases with distance to the river from 40% to 7%, while the silt and clay content increase (silt: 44% to 69%; clay: 16% to 24%). Experimental plots were arranged in four blocks parallel to the river to account for these differences in soil characteristics. Before the establishment of the Jena Experiment in 2002, the site was used as arable land since the early 1960s and ploughed and fertilized regularly. The Jena Experiment comprises 86 plots (82 vegetated and 4 bare ground plots, each 20 m by 20 m). The experimental design manipulates a gradient in sown plant species richness from 1 to 60 (1, 2, 4, 8, 16, and 60) near-orthogonal with a gradient in plant of functional group numbers from 1 to 4 (1, 2, 3 and 4). All 60 species are typical for Central European mesophilic grasslands [34], [35]. They were grouped into four functional groups according to their morphological, phenological and physiological traits. The species pool included 16 grasses, 12 small herbs, 20 tall herbs and 12 legumes [34]. To maintain the diversity levels, all experimental communities have been weeded manually twice a year. Plots are mown twice a year, in June and September and are not fertilized. In addition bare ground plots with four replicates were established. Furthermore, soil microbial community was determined on two adjacent regularly mown non-fertilized meadows and two arable plots on the experimental site. The arable plots were continuously managed according to conventional agricultural procedures, growing cereals.

### Soil sampling and phospholipid fatty acid (PLFA) analysis

In early May 2007, six soil samples per plot were taken with a core cutter (inner diameter: 4.8 cm, Eijkelkamp Agrisearch Equipment, Giesbeek, The Netherlands) to a depth of 5 cm, pooled and placed immediately in cooling boxes. Within 48 hours after sampling the soil was kept at 4°C, sieved <2 mm, remains of roots were manually removed and finally the samples were stored at −20°C until further sample processing. Soil samples were shaken with a mixture of chloroform, methanol and 0.05 M phosphate buffer (pH 7.4) to extract soil lipids [29], [36]. The lipids were split into neutral lipids, glycolipids and phospholipids by eluting with chloroform, acetone and methanol from a silica-filled solid phase extraction column. Subsequently, phospholipids were hydrolyzed and methylated by a methanolic KOH solution and the PLFA-methyl esters were identified and quantified by gas chromatography with atomic emission detector (GC-AED) (Agilent, Böblingen, Germany) and gas chromatography-mass spectrometry (GC/MS) (Thermo Electron, Dreieich, Germany). Peak areas and the resulting amount of PLFA were calculated relative to the internal standard PLFA 19:0. The sum of all PLFAs (Table S1) was taken as total soil microbial biomass. Furthermore PLFAs were assigned to microbial groups [37], [38]. The PLFAs 14:0, 14:0br, 15:0, 16:0, 17:0, 18:0 were used as general microbial markers. All monounsaturated and cyclic fatty acids were grouped as Gram-negative bacteria (Gram-), while all branched PLFAs were grouped as Gram-positive bacteria (Gram+). PLFA 18:2ω6 was used as a fungal biomarker [38], [39]. The F:B ratio was calculated using the molar weight of the fungal PLFA marker divided by the sum of molar weights of bacterial PLFA biomarker [40].

### Covariables

Fine root standing biomass (termed as ‘root biomass’ hereafter), leaf area index (LAI) and soil moisture were considered as potentially meaningful covariables. Unfortunately, in 2007, the year of the PLFA sampling, root biomass was not determined, thus we used an average of 2006 and 2008 root biomass measurements. In both years root biomass was sampled to a depth of 30 cm. In addition, the sampling in 2008 was stratified, so that the 0–5 cm depth increment could directly related to the sample of the soil microbes. Based on the ratio of the top increment (0–5 cm) to the total root biomass in 2008, we calculated the specific root biomass in the top soil (0–5 cm). Furthermore, nitrogen concentration of fine roots was determined using root material from ingrowth cores from, sampled between 2007 and 2008 [41]. N concentrations in the biomass were determined with an elemental analyzer (Vario EL Element Analyzer, Elementar, Hanau, Germany). In the course of the PLFA soil sampling soil moisture was determined, too, as the gravimetric soil water content. Leaf area index (LAI) was measured approx. 5 cm above ground level [42] using a LAI-2000 plant canopy analyzer (LI-COR) in late May 2007 (shortly before the first mowing of the year; see experimental design).

### Statistical analysis

Using analyses of variance (ANOVA) followed by Tukey's HSD test we assessed microbial biomass and F:B ratio in experimental plots of the Jena Experiment, and their relationship to those from control plots (arable fields and meadows). ANOVA with sequential sum of squares (type I SS) was applied to test for effects of plant diversity on microbial biomass (total, Gram+, Gram− and fungal). The Jena Experiment is based on a factorial design with different combinations of plant species richness and number of functional groups, where all plots are arranged in a block design accounting for differences in soil texture among the blocks [34]. Therefore, ‘block effect’ was included as random factor and was fitted first. The contrast between bare ground plots vs. sown plots was fitted next, before testing for the effect of richness (log-linear term) and functional groups (linear term) as continuous variables. Finally, the presence of each plant functional group (small herbs, tall herbs, grasses and legumes) was included into the model in a series of alternative models. Furthermore, non-metric multidimensional scaling (NMDS) [43] was used to compare plot-specific patterns of PLFA profiles. The data used in the NMDS was normalized to the peak area of the highest peak (18.1n11) set at 100%. Bray-Curtis was used as dissimilarity index.

To investigate which mechanisms underlie the effects of plant diversity, we used structural equation modelling (SEM, see also Table S2) with observed variables [44]. In SEM, all diversity levels, except bare ground, were considered. For every group of PLFAs assigned to specific microbial taxa, a full model was set up (Figure S1) including all experimental variables that were significant in the preceding ANOVA. As possible means by which the effect of plant diversity might be manifest, we included root biomass as measure of belowground plant input, root nitrogen concentration as measure of litter quality and LAI as a measure of plant community influence on evaporation and thus the microclimatic conditions (e.g., soil moisture and temperature). The categorical variable ‘block’ was substituted by the continuous variable ‘clay’ content of soil. We considered aboveground plant inputs as negligible, because all above ground biomass was harvested twice a year. The minimal parsimonious models were identified using specification search, based on the Bayes information criterion (BIC) [45]. The adequacy of the model was tested with Chi-squared tests (*χ^2^* tests) and root mean square error of approximation (RMSEA) [44].

ANOVA and Tukey's HSD test were performed using R 2.15.2 [46] and structural equation modeling was performed using AMOS 18.0 [47].

## Results

### Soil microbial biomass

**Figure 1:**
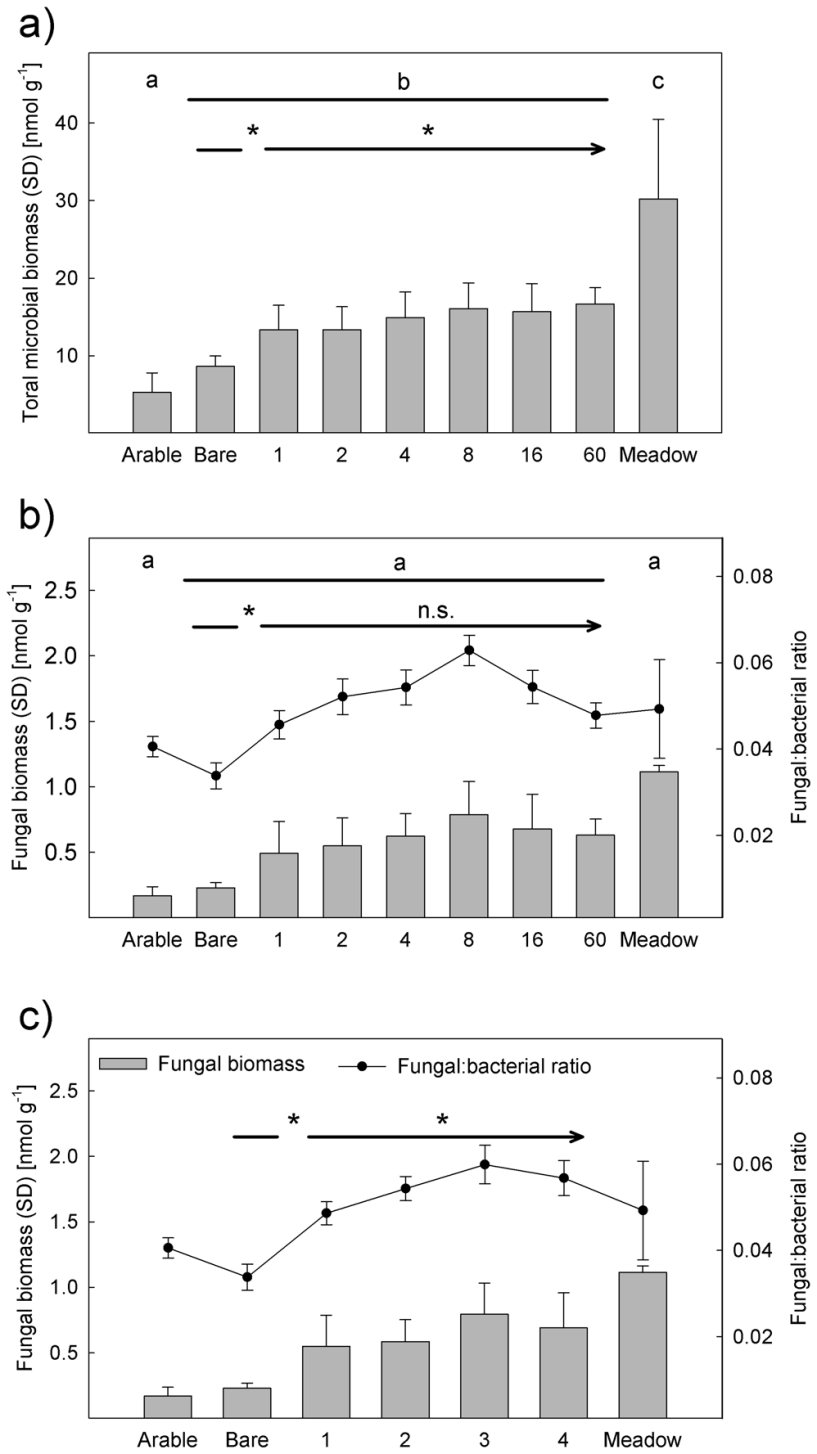
Impact of land use and plant diversity soil microbial community. Differences (*P*<0.05) between experimental plots and control sites (arable land and semi-natural meadows) were analyzed with Tukey's HSD test and indicated by letters. Differences between bare ground vs. vegetated plots and significant effects of plant diversity were tested with ANOVA (Table 1) and indicated by asterisks (*P*<0.05). Figures show effect of plant species richness on (a) total microbial biomass (b) fungal biomass and the fungal-to-bacterial biomass ratio and (c) number plant functional groups effect on fungal biomass and the fungal-to-bacterial biomass ratio.

The mean of the total PLFA concentration, henceforth termed total microbial biomass, was14.4±3.5 nmol g^−1^ soil dry weight (mean ± sd) on experimental plots (vegetated plots and bare ground). This was significantly higher than measured on plots of arable land (5.3±2.5 nmol g^−1^) and significantly lower than on meadows (30.2±10.3 nmol g^−1^; Figure 1a).

ANOVA revealed block as a significant predictor of the total microbial biomass (Table 1). Furthermore, total microbial biomass was significantly lower on bare ground plots (8.7±1.3 nmol g^−1^) than on vegetated plots (14.7±3.4 nmol g^−1^). Plant species richness had a significant positive effect on the total microbial biomass on vegetated plots (Table 1; Figure 1a). The presence of individual plant functional groups did not affect total microbial biomass.

**Table 1:** Results of ANOVAs on the effect of the experimental variables on microbial community.

		MicBM		F:B ratio		Fungi		Gram+		Gram-	
	Df	F value	*P*	F value	*P*	F value	*P*	F value	*P*	F value	*P*
Block	3	**9.33**	**<0.001**	**3.54**	**0.019**	**8.20**	**<0.001**	**8.64**	**<0.001**	**6.64**	**<0.001**
Bare grounds	1	**18.83**	**<0.001**	**8.93**	**0.004**	**16.10**	**<0.001**	**15.30**	**<0.001**	**18.19**	**<0.001**
PSR	1	**13.60**	**<0.001**	0.09	0.768	2.65	0.108	**8.70**	**0.004**	**13.84**	**<0.001**
FG	1	0.03	0.858	**6.72**	**0.012**	**11.70**	**<0.001**	0.00	0.968	0.00	0.974
legumes	1	0.00	0.983	**21.74**	**<0.001**	**9.83**	**0.002**	0.01	0.933	0.26	0.609
Grasses	1	1.62	0.207	1.83	0.180	0.08	0.775	**4.83**	**0.031**	0.93	0.339
Tall herbs	1	0.10	0.757	0.97	0.329	0.13	0.725	0.27	0.607	0.09	0.763
Small herbs	1	0.47	0.496	2.72	0.103	0.41	0.524	1.33	0.252	0.45	0.506

Impact of plant diversity (plant species richness (PSR, log transformed) and number plant functional groups (FG)) and presence of distinct plant functional groups (legumes, grasses, small herbs, tall herbs) on total microbial biomass (MicBM), Gram-positive (Gram+), Gram-negative bacteria (Gram−), fungal biomass (Fungi) and the composition of the soil community, characterized by the fungal-to-bacterial biomass ration (F:B ratio). Numbers in bold display p values < 0.05 and numbers in italic display p values <0.1.

**Figure 2:**
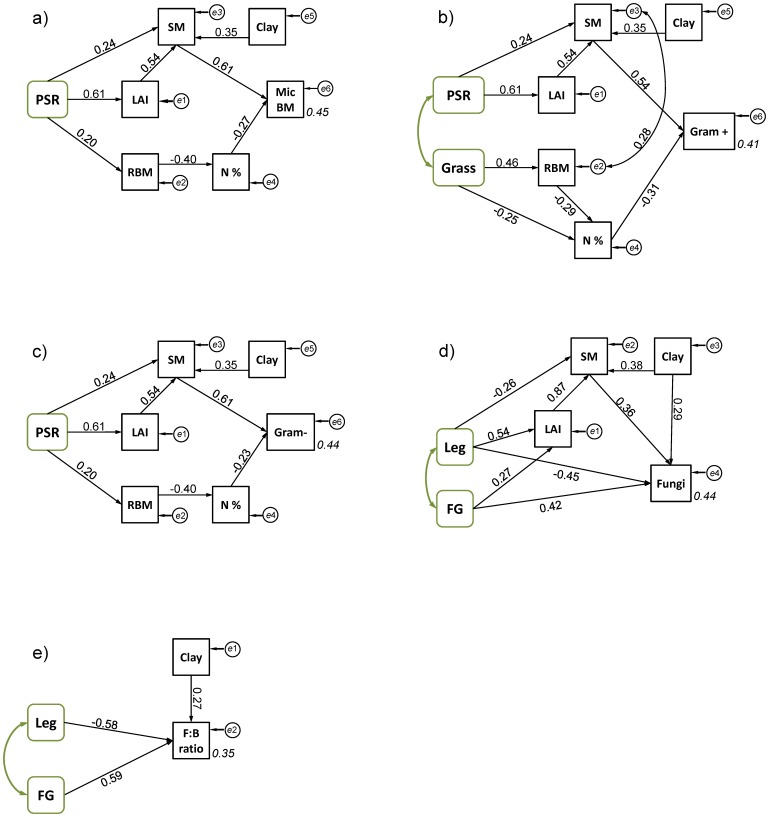
Minimal parsimonious models, testing direct and indirect effects of plant diversity on soil microbial community. Minimal SEM for a) total soil microbial biomass (MicBM), b) biomass of Gram positive bacteria (Gram+), c) biomass Gram negative bacteria (Gram−), d) fungal biomass (Fungi), and e) fungal-to-bacterial biomass ratio (F:B ratio). Arrows show significant relationships between variables. Numbers next to arrows show standardized parameter estimates (i.e., standardized regression weights). Circles (e1–e6) indicate error terms, and double-headed arrows indicate significant correlations between the error terms. Squared multiple correlations (*R^2^*) for the dependent soil microbial biomass are given next to the box of the dependent variable. See the non-standardized estimates of the regression weights in Table S3a-e. Abbreviations are PSR: plant species richness, FG: plant functional group richness, LEG: presence of legumes, GRASS: presence of grasses, RBM: fine root standing biomass, N%: nitrogen concentration of fine roots, LAI: leaf area index, SM: soil moisture, Clay: clay content of soil

Structural equation modeling (SEM) showed that block (represented by the continuous variable clay content of soil) and plant richness, the design variables with significant influence indirectly affected total microbial biomass (Figure 2a). The minimal parsimonious model (*χ^2^*_13_ = 21.74, *P* = 0.060; RMSEA = 0.093, *P* = 0.147) explained 45% of the variance of total microbial biomass. Total microbial biomass was mainly explained by its positive relationship to soil moisture. Soil moisture increased with increasing plant richness and higher clay content. The major effect on soil moisture was attributed to increasing leaf area index (LAI), which itself was strongly correlated to plant richness. The negatively influence of root nitrogen concentration on total microbial biomass was driven by higher root biomass, which itself was increased at higher plant richness.

### Soil microbial community structure

The F:B ratio did not differ among experimental plots, arable plots and meadow plots (experimental plots: 0.052±0.015; arable land: 0.041±0.003; meadows: 0.049±0.016; Figure 1b). In contrast, the F:B ratio was significantly lower on bare ground (0.034±0.006) than on vegetated plots of the biodiversity experiment (0.053±0.015). F:B ratio was positively affected by an increasing number of plant functional groups and negatively by the presence of legumes (Table 1; Figure 1c). Plant richness did not significantly affect the F:B ratio. However, the F:B ratio increased from low plant richness plots to medium ones with eight plant species, and decreased again in plots with high plant richness. Regression analyses of the relationship between F:B ratio and both, fungal and bacterial biomass revealed that the F:B ratio was more related to fungi (R^2^ = 0.67, *P*<0.001) than to bacterial biomass (R^2^ = 0.043, *P*<0.066).

Considering biomass of Gram+, Gram- and fungi separately, all groups differed significantly among blocks and between bare ground and vegetated plots (Table 1). Plant diversity positively affected both bacterial groups. Gram+ as well as Gram- bacteria were reduced on bare ground plots (Gram+ = 2.3±0.4; Gram- = 4.6±0.6) compared to vegetation plots (Gram+ = 3.9±1.0; Gram- = 7.7±1.7) and biomass of both bacterial groups were increased with increasing plant richness (Table 1). However, the ANOVA also revealed differences: while Gram+ were positively influenced by the presence of grasses, Gram- were not affected by any of the plant functional groups. Fungal biomass was positively affected by the number of functional groups present, and negatively by the presence of legumes.

**Figure 3:**
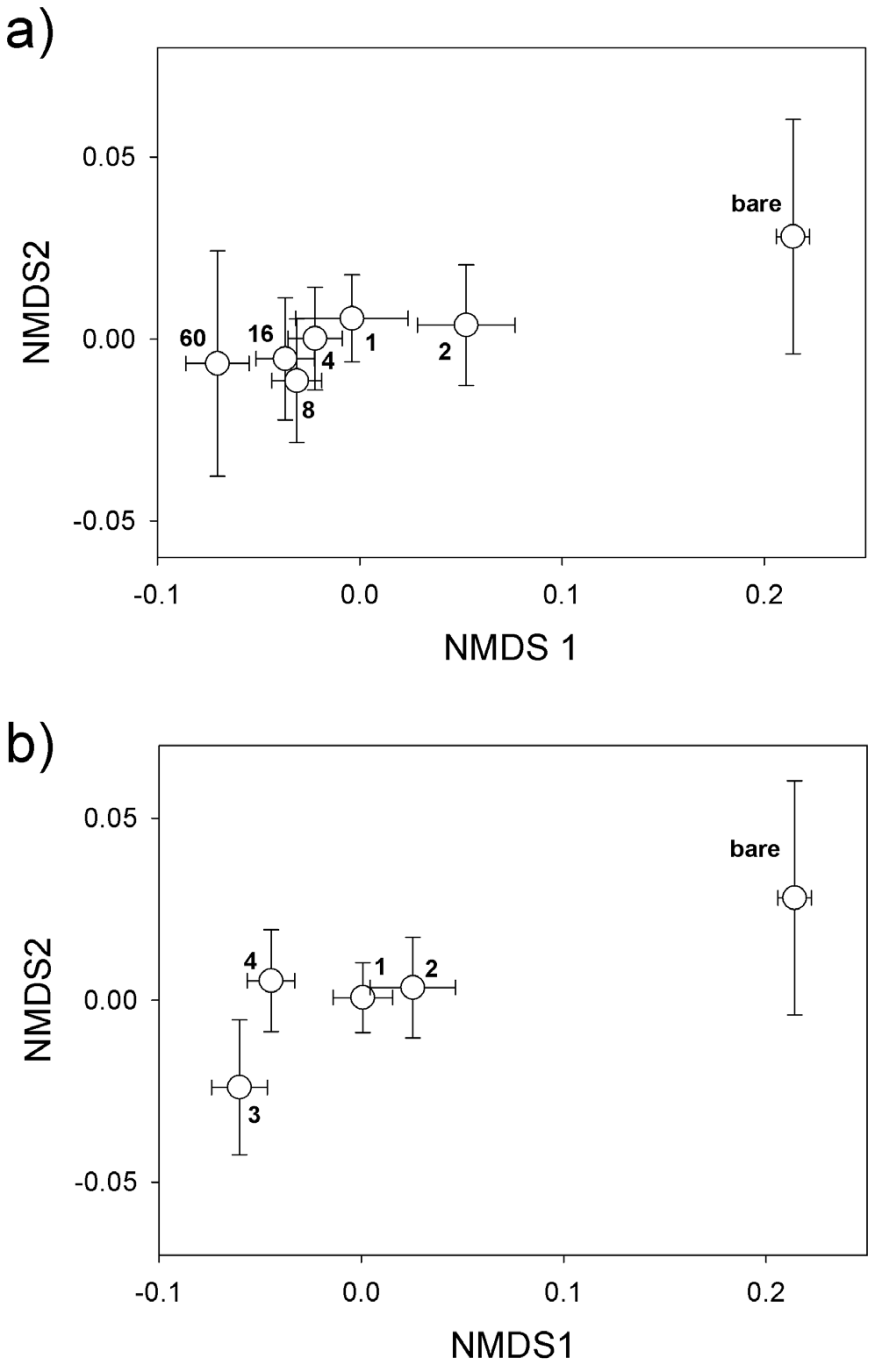
Summary of non-metric multidimensional scaling (NMDS) of the PLFAs. Differences among bare ground plots and different plant diversity levels are shown for (a) 1 to 60 sown plant species and (b) 1 to 4 functional groups. Bray-Curtis was used as dissimilarity index.

The results of the NMDS (Figure 3), based on the PLFA composition, confirmed the strong dissimilarity between bare ground plots and vegetated plots. The dissimilarity between the vegetated plots was relatively small, though we found a clear effect of plant diversity, i.e. the higher the plant diversity on the plot the more different were the microbial communities compared to low diverse plots.

SEM for Gram+ (*χ^2^*_17_ = 24.877, *P* = 0.098; RMSEA = 0.078, *P* = 0.231) and Gram- bacteria (*χ^2^*_13_ = 19.80, *P* = 0.100; RMSEA = 0.082, *P* = 0.217) explained to 41% and 44% of variance, respectively, and revealed high analogy between the groups (Figure 2b, 2c). Both bacterial groups were mainly driven by soil moisture, which was mostly affect by LAI. Furthermore, both groups had a negative relationship with nitrogen concentration of fine roots, mediated by increased root biomass. The minimal parsimonious model explains 44% the variation in fungal biomass (*χ^2^*_5_ = 5.43, *P* = 0.365; RMSEA = 0.034, *P* = 0.475). In contrast to the bacterial groups, fungal biomass was neither affected by the amount of root biomass nor by its quality (Figure 2d). Although there was a positive indirect pathway from functional groups and legumes via LAI and soil moisture to fungal biomass, strong direct paths from functional groups and legumes remained in the minimal parsimonious model. These direct paths indicate that the diversity effect was driven by mechanisms other than soil moisture or quantity and quality of root biomass. The F:B ratio was explained to 35% by the minimal model (*χ^2^*_2_ = 2.27, *P* = 0.322; RMSEA = 0.041, *P* = 0.390). In contrast to all other models, only direct paths connected the experimental variables to F:B ratio (Figure 2e): it strongly decreased in the presence of legumes, but increased with increasing number of plant functional groups to almost the same extent. These relationships could not be explained by our measured covariables.

## Discussion

In the framework of the Jena Experiment we investigated how soil microbial communities are affected by plant diversity and the underlying mechanisms of these effects. In grassland with manipulated plant species richness and number of plant functional groups we showed that the soil microbial communities are strongly linked to plant diversity. Corresponding to hypothesis 1, the positive plant diversity effect on total microbial biomass was mainly driven by improved microclimatic conditions, while we found only a minor influence of the amount of root litter inputs on the soil microbes. Furthermore, number of plant functional groups and the plant functional composition, in particular the presence of legumes, highly impact the microbial community composition, referring to hypothesis 2. Below, we will discuss in detail, how plant diversity drives the soil microbial community.

Five years after conversion from arable land to grasslands, increased soil microbial biomass indicates that the microbial community performs better. In addition to the growth of the soil microbial community, it has been reported that the community also has a higher metabolic activity compared to the initial conditions [21]. Lower microbial biomass on arable land probably is due to soil disturbance by tillage and the tillage-induced changes of soil properties [48]. The lower organic carbon concentration in arable soils is attributed to faster decomposition of soil organic matter, which in turn reduces the microbial biomass in the long term (e.g., [49], [50]). However, even in plots with highest plant diversity, i.e., 60 species and 4 functional groups, microbial biomass was lower than in adjacent meadows. This indicates that the time since conversion of our study area from arable use to grassland was not sufficient to reach the state of microbial biomass of permanent meadows. However, as total microbial biomass significantly increased with increasing plant richness, higher plant diversity promotes the development towards the stage of permanent meadows (Figure 1a).

Confirming hypothesis 1, plant richness as well as clay content of the soil indirectly increased total soil microbial biomass. Interestingly, structural equation modeling suggests that this was mediated via soil moisture. Soil moisture itself holds a central position in the interplay between plant diversity, abiotic soil conditions and microbial biomass (Figure 2a). The strong influence of soil texture on soil moisture is well known: with smaller particle size soil water holding capacity increases. Results of the present study suggest, however, that the positive effect of plant diversity on soil microbial biomass may exceed that soil texture via changes in soil moisture. Higher plant diversity increased canopy density of the plant stands, measured as LAI, which presumably reduced evaporation from the soil surface [23], [51]. Plant richness also affected soil microbial biomass via root inputs, namely via root nitrogen concentration. The detrimental effect of nitrogen concentration on microbial biomass was closely related to increased root biomass; with increasing plant richness root biomass increased, while at the same time nitrogen concentration decreased, which confirms earlier findings [22], [41].

Results of NMDS showed that the composition of PLFAs differed mainly between bare ground and vegetated plots, while in vegetated plots (1-60 plant species, 1-4 plant functional groups) the composition of PLFAs was similar. However, the dissimilarity of the microbial community composition was more pronounced in plots with different diversity levels, i.e., low diverse plots differed most from high diverse plots. Higher diversity in plant communities leads to more diverse organic matter input in quantity, quality and timing [9], [13], and this likely is responsible for the observed changes in microbial communities along diversity levels. The plant diversity effect on microbial community composition was also reflected in the F:B ratio, but in contrast to total microbial biomass, the F:B ratio was more affected by functional groups than by species richness, supporting hypothesis 2. Moreover, this relation to plant functional groups reflects the stronger dependency of F:B ratios from changes in fungal biomass than in bacterial biomass [52]. We further found legumes to be a strong predictor of F:B ratio, which is in line with previous findings [15], [24], [53]. However, neither the underlying mechanisms of the positive effect of functional groups nor of the negative legume effect on the F:B ratio was mediated by the considered covariables. Similar results have been reported by Lamb *et al.* [54], who studied the effect of plant species richness and evenness on soil microbial communities in a pot experiment. The lack of relationships between F:B ratio and root litter quantity and quality as well as soil moisture indicates that both microbial groups are similarly affected by these variables. The strong direct link between plant diversity and F:B ratio, however, points to other plant resources as major drivers of soil microorganisms, such as root exudates. Indeed, root exudates were reported to strongly influence soil microbial communities [14], [55]. In more diverse plant mixtures resource supply for microorganisms may be assumed to be higher and more diverse, while resource supply in monocultures is expected to be more one-sided and temporally limited. Furthermore, it is known, that the number of plant functional groups and presence of legumes may be related to turnover rates and decomposition of fine roots [24], [56], [57], which might cause changes in microbial community structure.

Although bacteria and fungi were similarly affected by plant diversity, we found bacteria more related to plant diversity-controlled abiotic soil properties, while and fungi were more affected by the input of organic materials. As shown by de Vries *et al.* [58] fungal-based soil food webs are more resistant to disturbances, while bacterial communities are more resilient due to their fast life cycle. This might explain why the bacterial community was in our study more related to fast changing abiotic conditions, such as soil moisture.

## Conclusion

We identified changes in microclimatic conditions, in particular increased soil moisture, as a main mechanism how plant diversity affects soil microbial biomass in the topsoil. Furthermore, the results indicate that shifts in the microbial community composition, namely in the F:B ratio, heavily rely on differences in the quality and quantity of root exudates. Changes in soil microbial biomass with plant diversity suggest that microbial communities of the established grassland systems develop towards permanent meadows, but that reaching the state of these meadows takes decades. Notably, however, differences in microbial biomass indicate that high diverse plant communities promote faster transition towards permanent meadows indicating that plant diversity is a key factor for restoring functional grassland systems on former arable land.

## Supporting Information

Figure S1 Scheme of the full model used in the structural equation modelling. The full model included simultaneously all measures of plant diversity with significant impact (potentially plant species richness, number of plant functional groups and the presence/absence of legumes, grasses, small herbs and tall herbs) on microbes. Measurements of plant inputs (fine root biomass (RBM), and nitrogen content of fine roots (N%) leaf area index (LAI), soil moisture (SM) and clay content of soil (Clay) have also been included in the model to explain the underlying mechanisms of the diversity effect.
(TIF)

Table S1 Mean concentrations of identified PLFAs in nmol g^−1^ dw (dry weight).
(DOCX)

Table S2 Correlation matrix of the predictors. Abbreviations are: Block numeric (Bl_num), logarithmic plant species richness (log_PSR), number of plant functional groups (FG), presence/absence of grasses (gras), presence/absence of small herbs (sherb), presence/absence of tall herbs (therb), presence/absence of legumes (leg), soil content of clay [%] (clay), leaf area index (LAI), fine root standing biomass [g m^−2^] (RBM), nitrogen content of fine root biomass [%] (N%).
(DOCX)

Table S3 Estimates of the minimal adequate structural equation models (maximum likelihood) for a) Total microbial biomass (MicMB b) Gram positive bacteria (Gram+), c) Gram negative bacteria (Gram-), d) Fungal-to-bacterial ratio (F:B ratio) and e) Fungi.
(DOCX)
