# Supplementary figures and images for: Biotic and Abiotic Properties Mediating Plant Diversity Effects on Soil Microbial Communities in an Experimental Grassland
Source: PLoS One. 2014 May 9;9(5):e96182. doi: 10.1371/journal.pone.0096182 (PMC4015938; doi:10.1371/journal.pone.0096182)

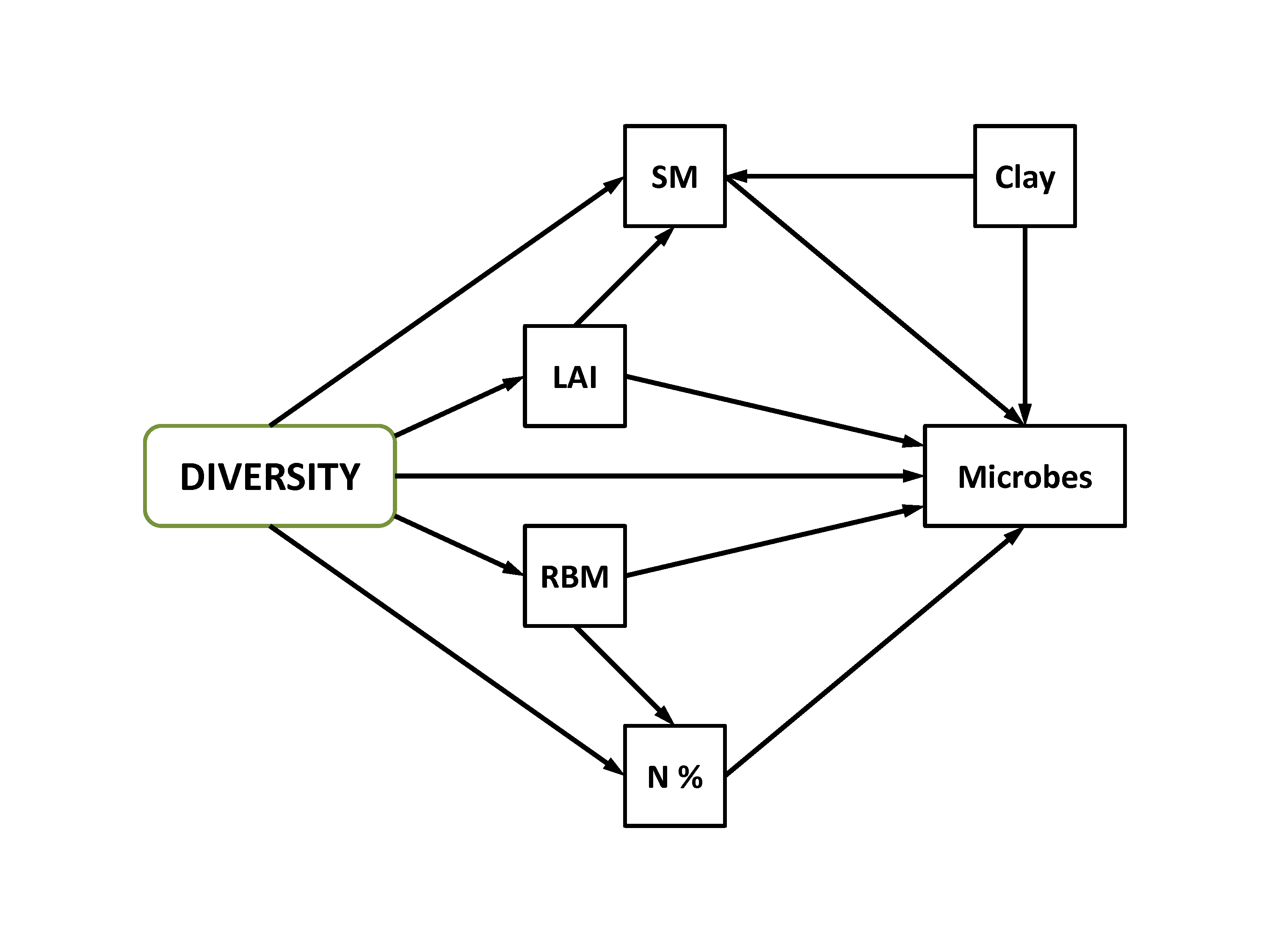

Supplement: Figure S1 — Scheme of the full model used in the structural equation modelling. The full model included simultaneously all measures of plant diversity with significant impact (potentially plant species richness, number of plant functional groups and the presence/absence of legumes, grasses, small herbs and tall herbs) on microbes. Measurements of plant inputs (fine root biomass (RBM), and nitrogen content of fine roots (N%) leaf area index (LAI), soil moisture (SM) and clay content of soil (Clay) have also been included in the model to explain the underlying mechanisms of the diversity effect. (TIF) [file pone.0096182.s001.tif]
