# Supplementary material for: Biotic and Abiotic Properties Mediating Plant Diversity Effects on Soil Microbial Communities in an Experimental Grassland
Source: PLoS One. 2014 May 9;9(5):e96182. doi: 10.1371/journal.pone.0096182 (PMC4015938; doi:10.1371/journal.pone.0096182)
Supplement: Table S1 — Mean concentrations of identified PLFAs in nmol g−1 dw (dry weight). (DOCX) [file pone.0096182.s002.docx]

**Table S1:**

| **PLFA** | **Microbial group** | **Mean nmol g-1** | | | |
| --- | --- | --- | --- | --- | --- |
|  |  | arable | bare | vegetation-covered | meadow |
| 14:0br | general | 0.27 | 0.72 | 28.71 | 2.91 |
| 14:0 | general | 0.11 | 0.32 | 13.15 | 0.50 |
| 16:0 | general | 1.11 | 3.70 | 124.50 | 5.75 |
| 18:0 | general | 0.19 | 0.78 | 24.57 | 0.55 |
| 17:0 | general | 0.09 | 0.88 | 15.33 | 0.35 |
| 15:0br_i | gram + | 0.80 | 2.49 | 72.98 | 3.48 |
| 15:0br_a | gram + | 0.75 | 2.01 | 67.53 | 3.47 |
| 16:0br | gram + | 0.14 | 0.61 | 6.02 | 0.17 |
| 16:0br | gram + | 0.21 | 0.48 | 33.39 | 1.57 |
| 17:0br | gram + | 0.10 | 0.44 | 10.11 | 0.64 |
| 17:0br | gram + | 0.57 | 1.97 | 82.54 | 4.25 |
| 17:0br | gram + | 0.22 | 0.78 | 23.77 | 1.17 |
| 18:0br | gram + | 0.16 | 0.20 | 10.73 | 1.19 |
| 16:1 | gram - | 1.17 | 3.44 | 109.73 | 7.37 |
| 17:1 | gram - | 0.12 | 1.64 | 45.77 | 0.87 |
| 17:1 | gram - | 0.45 | 0.30 | 12.39 | 2.10 |
| 17:1 | gram - | 0.16 | 1.09 | 35.36 | 0.25 |
| 18:0_c | gram - | 0.58 | 1.85 | 55.97 | 4.24 |
| 18:1(n9) | gram - | 1.26 | 4.63 | 144.28 | 6.55 |
| 18:1(n11) | gram - | 1.57 | 4.64 | 182.07 | 9.33 |
| 18:1 | gram - | 0.20 | 0.74 | 25.45 | 1.49 |
| 18:2(n6) | fungal | 0.34 | 0.91 | 49.38 | 2.23 |
|  |  |  |  |  |  |
| Sum Gram+ |  | 2.95 | 8.99 | 307.06 | 15.94 |
| Sum Gram- |  | 5.52 | 18.32 | 611.01 | 32.20 |
| Sum Fungi |  | 0.34 | 0.91 | 49.38 | 2.23 |
