# Supplementary material for: Biotic and Abiotic Properties Mediating Plant Diversity Effects on Soil Microbial Communities in an Experimental Grassland
Source: PLoS One. 2014 May 9;9(5):e96182. doi: 10.1371/journal.pone.0096182 (PMC4015938; doi:10.1371/journal.pone.0096182)
Supplement: Table S2 — Correlation matrix of the predictors. Abbreviations are: Block numeric (Bl_num), logarithmic plant species richness (log_PSR), number of plant functional groups (FG), presence/absence of grasses (gras), presence/absence of small herbs (sherb), presence/absence of tall herbs (therb), presence/absence of legumes (leg), soil content of clay [%] (clay), leaf area index (LAI), fine root standing biomass [g m−2] (RBM), nitrogen content of fine root biomass [%] (N%). (DOCX) [file pone.0096182.s003.docx]

**Table S2:**

|  | Bl_num | log_PSR | FG | gras | sherb | therb | Leg | clay | LAI | RBM | N% |
| --- | --- | --- | --- | --- | --- | --- | --- | --- | --- | --- | --- |
| Bl_num | 1 |  |  |  |  |  |  |  |  |  |  |
| log_PSR | -0.01 | 1 |  |  |  |  |  |  |  |  |  |
| FG | 0.01 | 0.64 | 1 |  |  |  |  |  |  |  |  |
| Gras | -0.02 | 0.36 | 0.57 | 1 |  |  |  |  |  |  |  |
| sherb | 0.01 | 0.37 | 0.59 | 0.20 | 1 |  |  |  |  |  |  |
| therb | -0.01 | 0.37 | 0.54 | 0.15 | -0.08 | 1 |  |  |  |  |  |
| leg | 0.04 | 0.36 | 0.59 | -0.06 | 0.23 | 0.18 | 1 |  |  |  |  |
| clay | 0.81 | -0.08 | -0.04 | -0.11 | 0.07 | -0.17 | 0.11 | 1 |  |  |  |
| LAI | -0.04 | 0.61 | 0.59 | 0.23 | 0.16 | 0.26 | 0.70 | -0.04 | 1 |  |  |
| RBM | 0.17 | 0.20 | 0.09 | 0.44 | 0.13 | -0.08 | -0.27 | 0.20 | -0.05 | 1 |  |
| N% | -0.22 | -0.08 | -0.02 | -0.38 | 0.06 | 0.05 | 0.22 | -0.28 | 0.17 | -0.40 | 1 |
