# Supplementary material for: Biotic and Abiotic Properties Mediating Plant Diversity Effects on Soil Microbial Communities in an Experimental Grassland
Source: PLoS One. 2014 May 9;9(5):e96182. doi: 10.1371/journal.pone.0096182 (PMC4015938; doi:10.1371/journal.pone.0096182)
Supplement: Table S3 — Estimates of the minimal adequate structural equation models (maximum likelihood) for a) Total microbial biomass (MicMB b) Gram positive bacteria (Gram+), c) Gram negative bacteria (Gram-), d) Fungal-to-bacterial ratio (F:B ratio) and e) Fungi. (DOCX) [file pone.0096182.s004.docx]

**Table S3a:**

Regression Weights and Standardized Regression Weights:

|  |  |  | Estimate | S.E. | C.R. | P | standard.Estimate |
| --- | --- | --- | --- | --- | --- | --- | --- |
| LAI | 🡨 | PSR | 1.378 | 0.206 | 6.675 | <0.001 | 0.605 |
| RBM | 🡨 | PSR | 20.974 | 11.976 | 1.751 | 0.080 | 0.196 |
| SM | 🡨 | LAI | 0.801 | 0.129 | 6.227 | <0.001 | 0.543 |
| SM | 🡨 | PSR | 0.800 | 0.293 | 2.731 | 0.006 | 0.238 |
| SM | 🡨 | Clay | 0.307 | 0.061 | 5.033 | <0.001 | 0.349 |
| N% | 🡨 | RBM | -0.001 | 0.000 | -3.831 | <0.001 | -0.400 |
| Mic MB | 🡨 | N% | -5.722 | 1.809 | -3.164 | 0.002 | -0.267 |
| Mic MB | 🡨 | SM | 0.672 | 0.094 | 7.173 | <0.001 | 0.606 |

Squared Multiple Correlations:

|  | Estimate |
| --- | --- |
| Clay | 0.000 |
| LAI | 0.367 |
| RBM | 0.038 |
| SM | 0.630 |
| N% | 0.160 |
| Mic MB | 0.452 |

**Table S3b:**

Regression Weights and Standardized Regression Weights:

|  |  |  | Estimate | S.E. | C.R. | P | Estimate |
| --- | --- | --- | --- | --- | --- | --- | --- |
| LAI | 🡨 | PSR | 1.378 | 0.206 | 6.675 | <0.001 | 0.605 |
| RBM | 🡨 | grass | 91.031 | 19.293 | 4.718 | <0.001 | 0.459 |
| SM | 🡨 | LAI | 0.801 | 0.129 | 6.227 | <0.001 | 0.543 |
| SM | 🡨 | PSR | 0.800 | 0.293 | 2.731 | 0.006 | 0.238 |
| SM | 🡨 | Clay | 0.307 | 0.061 | 5.033 | <0.001 | 0.349 |
| N% | 🡨 | RBM | 0.000 | 0.000 | -2.586 | 0.010 | -0.294 |
| N% | 🡨 | grass | -0.079 | 0.036 | -2.185 | 0.029 | -0.248 |
| Gram+ | 🡨 | N% | -1.925 | 0.544 | -3.537 | <0.001 | -0.311 |
| Gram+ | 🡨 | SM | 0.170 | 0.028 | 6.031 | <0.001 | 0.530 |

Squared Multiple Correlations:

|  | Estimate |
| --- | --- |
| Clay | 0.000 |
| LAI | 0.367 |
| RBM | 0.210 |
| SM | 0.630 |
| N% | 0.215 |
| Gram+ | 0.412 |

**Table S3c:**

Regression Weights and Standardized Regression Weights:

|  |  |  | Estimate | S.E. | C.R. | P | Estimate |
| --- | --- | --- | --- | --- | --- | --- | --- |
| LAI | 🡨 | PSR | 1.378 | 0.206 | 6.675 | <0.001 | 0.605 |
| RBM | 🡨 | PSR | 20.974 | 11.976 | 1.751 | 0.080 | 0.196 |
| SM | 🡨 | LAI | 0.801 | 0.129 | 6.227 | <0.001 | 0.543 |
| SM | 🡨 | PSR | 0.800 | 0.293 | 2.731 | 0.006 | 0.238 |
| SM | 🡨 | Clay | 0.307 | 0.061 | 5.033 | <0.001 | 0.349 |
| N% | 🡨 | RBM | -0.001 | 0.000 | -3.831 | <0.001 | -0.400 |
| Gram- | 🡨 | N% | -2.462 | 0.911 | -2.703 | 0.007 | -0.231 |
| Gram- | 🡨 | SM | 0.338 | 0.047 | 7.169 | <0.001 | 0.612 |

Squared Multiple Correlations:

|  | Estimate |
| --- | --- |
| Clay | 0.000 |
| LAI | 0.367 |
| RBM | 0.038 |
| SM | 0.630 |
| N% | 0.160 |
| Gram- | 0.440 |

**Table S3d:**

Regression Weights and Standardized Regression Weights:

| **Fungi** |  |  | Estimate | S.E. | C.R. | P | Estimate |
| --- | --- | --- | --- | --- | --- | --- | --- |
| LAI | 🡨 | FG | 0.504 | 0.174 | 2.896 | 0.004 | 0.277 |
| LAI | 🡨 | LEG | 2.232 | 0.398 | 5.615 | <0.001 | 0.537 |
| SM | 🡨 | LAI | 1.290 | 0.144 | 8.972 | <0.001 | 0.865 |
| SM | 🡨 | Clay | 0.327 | 0.061 | 5.352 | <0.001 | 0.368 |
| SM | 🡨 | LEG | -1.628 | 0.598 | -2.724 | 0.006 | -0.263 |
| Fungi | 🡨 | Clay | 0.021 | 0.007 | 3.084 | 0.002 | 0.287 |
| Fungi | 🡨 | FG | 0.092 | 0.024 | 3.907 | <0.001 | 0.424 |
| Fungi | 🡨 | LEG | -0.222 | 0.054 | -4.136 | <0.001 | -0.446 |
| Fungi | 🡨 | SM | 0.029 | 0.008 | 3.506 | <0.001 | 0.356 |

Squared Multiple Correlations:

|  | Estimate |
| --- | --- |
| Clay | 0.000 |
| LAI | 0.541 |
| SM | 0.635 |
| Fungi | 0.439 |

**Table S3e:**

Regression Weights and Standardized Regression Weights:

|  |  |  | Estimate | S.E. | C.R. | P | Estimate |
| --- | --- | --- | --- | --- | --- | --- | --- |
| F:B ratio | 🡨 | Clay | 0.003 | 0.001 | 2.917 | 0.004 | 0.268 |
| F:B ratio | 🡨 | FG | 0.018 | 0.003 | 5.166 | <0.001 | 0.587 |
| F:B ratio | 🡨 | LEG | -0.040 | 0.008 | -5.111 | <0.001 | -0.581 |

Squared Multiple Correlations:

|  | Estimate |
| --- | --- |
| Clay | 0.000 |
| F:B ratio | 0.351 |
